# Differences between Goldmann Applanation Tonometry and Dynamic Contour Tonometry following Trabeculectomy

**DOI:** 10.1155/2010/357387

**Published:** 2010-07-13

**Authors:** Efstathios T. Detorakis, Emilia Grammenandi, Ioannis G. Pallikaris, Miltiadis K. Tsilimbaris

**Affiliations:** Department of Ophthalmology, University Hospital of Heraklion, 71110 Heraklion, Crete, Greece

## Abstract

*Background*. To evaluate differences between Goldmann Applanation Tonometry (GAT) and Dynamic Controur Tonometry (DCT) following trabeculectomy. *Methods*. Thirty eight glaucomatous eyes with a history of trabeculectomy (Trabeculectomy group, TG), 20 eyes without a history of trabeculectomy but with a history of latanoprost use (Latanoprost group, LG), and 19 nonglaucomatous eyes (Control group, CG) were included. GAT-IOP, DCT-IOP, the difference between them (dIOP), the central corneal thickness (CCT), the axial length (AL), and the depth of the anterior chamber (ACD) were measured. *Results*. dIOP was significantly higher in TG (5.19 mmHg) than in LG (4.01 mmHg) and CG (1.98 mmHg). Correlations between AL and dIOP were statistically significant in both TG and LG but not in CG whereas correlations between dIOP and other clinical parameters examined were statistically not significant in all groups. *Conclusions*. The significantly higher dIOP in TG implies that the bio-mechanical properties of the ocular walls are altered following trabeculectomy.

## 1. Introduction

Differences in the measurements of the intraocular pressure (IOP) by different tonometry methods, such as Goldmann Applanation Tonometry (GAT) and Dynamic Contour Tonometry (DCT), have been previously detected in glaucomatous eyes under treatment with latanoprost and have been used as indicators of the biomechanical behaviour of the eye [1, 2]. Perhaps the most important determinator of the latter is ocular rigidity, corresponding to the mathematical relationship between pressure and volume changes of the eye [3, 4]. Ocular rigidity may be altered by conditions affecting the structural integrity of the ocular walls, such as trauma or surgery [5]. In the case of trabeculectomy, a partial thickness scleral flap (varying in depth or surface area) is created adjacent to the corneoscleral limbus, potentially modifying the biomechanical properties of the ocular walls [5–7]. This study aims at evaluating the difference between GAT and DCT (dIOP) in glaucomatous eyes treated with trabeculectomy, in glaucomatous eyes under topical treatment with latanoprost as well as in a control group of non-glaucomatous eyes and at correlating results with clinical information. Findings could help in assessing the potential effects of trabeculectomy on ocular rigidity and its possible role in the long-term modification of the clinical behaviour of glaucoma.

## 2. Materials and Methods

This is a prospective nonrandomized cohort study comparing three groups of subjects: a post-trabeculectomy group, a latanoprost group, and a normal control group. All patients included were Caucasians, consecutively recruited from the Department of Ophthalmology of the University Hospital of Heraklion, in Crete, Greece. Patients with a history of cataract or refractive surgery, trauma or inflammation in either eye were excluded to avoid potential effects of different intraocular lens implants (IOLs) or aphakia as well as different wound healing responses on measurements. The Trabeculectomy Group (TG) included glaucomatous patients with a history of 1 or 2 trabeculectomies in at least 1 eye. In all eyes of the TG, the target intraocular pressure (IOP) had been achieved, without any medication. The setting of a target IOP had been based on the formula proposed by Jampel: “Target IOP = Maximum initial IOP *(1 − Maximum initial IOP/100) − Z”, where Z is an optic nerve damage severity factor graded from 0 (for glaucoma suspects) to 5 (for end-stage glaucoma) [8]. The Latanoprost Group (LG) included patients with glaucoma in both eyes in whom target IOP (as previously mentioned) had also been achieved in at least 1 eye using latanoprost eye drops (as monotherapy) without a history of previous ocular surgery. The Control Group (CG) included cataract surgery candidates in whom glaucoma had been excluded in both eyes, who had no previous history of ocular surgery or trauma and who received no ocular medications. In all TG and LG eyes, target IOP had been reached at least 5 months prior to recruitment. The presence or absence of glaucoma was separately examined by 2 independent experienced examiners (ETD and MKT) and only patients with consent from both examiners were included. Criteria used for glaucoma diagnosis included IOP measurements consistently above 21 mmHg, a cup-to-disk ratio above 0.5 and in automated perimetry (with central 30-2 threshold test, Humphrey Field Analyzer/HFA II-I, 30-2, Carl Zeiss-Meditec Inc., Dublin, Calif, USA), and a Pattern Standard Deviation (PSD) score outside 95% limits of the normal reference. In all groups only one eye was enrolled per patient. In the case of TG patients with an equal number of trabeculectomies for both eyes, only the right eye was enrolled otherwise the eye with the greater number of procedures was enrolled. In the case of LG with latanoprost monotherapy in one eye and additional topical medications in the fellow eye, only the eye with latanoprost monotherapy was enrolled whereas in case of latanoprost monotherapy in both eyes only the right eye was enrolled. In the case of CG, only the right eye was enrolled. All patients signed a written informed consent form in accordance with the tenets of the Declaration of Helsinki.

All primary trabeculectomies were unenhanced (performed without the use of adjunctive intraoperative antimetabolites) and all had been performed by the same surgeon (ETD). The procedure was performed in all cases under topical anesthesia which included proparacaine eye drops and the injection of 2 ml of ropivacaine subconjunctivally. The latter was used to both anesthetize the area of flap creation (which in primary cases was always at the 12 o'clock position of the corneoscleral limbus) and hydro-dissect the conjunctiva from the underlying Tenon's capsule. A radial conjunctival incision was performed in all cases to facilitate exposure of the scleral bed, followed by an incision along the corneoscleral limbus to create a fornix-based conjunctival flap. Wide subconjunctival dissection was then performed followed by removal of any remaining Tenon's capsule overlying the area of scleral flap. A 4 × 4 mm partial thickness scleral flap was then marked (using monopolar diathermy) and created (using a 15° angled blade and a beaver blade) until the plane of dissection reached clear cornea (anteriorly to the scleral spur). At this point, a side port was created and then the anterior chamber was entered with a 15° blade anteriorly to the scleral spur. Trabeculectomy and iridectomy were performed (using a 0.75 mm corneoscleral punch and Vannas scissors, resp.), and the scleral flap was closed with 2 nylon sutures (10.0). The patency of trabeculectomy was tested by injecting balanced salt solution from the side port and observing the outflow from the trabeculectomy site and at that point adjustment of suture tying was performed as needed. The conjunctiva was then closed with 2 tight vicryl sutures (7.0) at the ends of the fornix-based flap forcing the conjunctiva firmly against the scleral bed to effectively seal the wound along the limbus. The remaining radial incision was also sutured with 7.0 vicryl sutures. In case a secondary trabeculectomy was performed for failed primary procedures (for patients who necessitated oral acetazolamide to control the IOP), the procedure was repeated in the same way, always nasally to the initial site (at the superior-nasal conjunctiva) to preserve the superior-temporal quadrant for possible future antiglaucomatous valve implantation. In all cases of a secondary trabeculectomy, Mitomycin-C 0.2% was also used (applied episclerally for 2 min).

GAT-IOP (mmHg), DCT-IOP (mmHg), dIOP, Central Corneal Thickness (CCT, *μ*m), AL (mm), and the anterior chamber depth (ACD) were examined in all patients by an experienced examiner (EG) who was masked against group classification. The latter had been performed previously and had been based on examinations evaluated by other examiners (ETD and MKT). The number or previous trabeculectomies in the TG (1 or 2) and the postoperative interval following the last trabeculectomy (in months) were also recorded. In the case of TG and LG, the peri-papillary nerve fiber thickness was also measured with GDx-VCC (Carl Zeiss Meditec, Dublin, Calif, USA) and the universal Nerve Fiber Index (NFI) was recorded. In the case of DCT (SMT Swiss Microtechnology AG, Port, Switzerland), the mean value of 3 readings of good quality, that is, Q1-Q3, as recommended by the manufacturer, was recorded. GAT was performed at least 10 minutes after DCT. The difference between GAT-IOP and DCT-IOP (dIOP) was then calculated. CCT, ACD, and AL were examined with the Alcon OcuScan RxP Ophthalmic Ultrasound System (Alcon laboratories, Alcon, Irvine, Calif, USA). A 20 Mhz probe was used for pachymetry (with a resolution of ±1 *μ*m and an accuracy of ±5 *μ*m) and a 10 Mhz probe was used for biometry (with a resolution of ±0.1 mm and a theoretical accuracy of ±0.05 mm). Ten successive measurements for AL, CCT, and ACD were taken in all cases and the mean was recorded. All clinical ophthalmic examinations were performed by the same experienced examiner (EG) who was masked against the classification of participants into TG, LG, and CG. 

The TG included 38 eyes of 38 patients (21 males, 55.26%), aged 71.19 ± 5.70 (55–84) years (mean ± SD, range). The LG included 20 eyes of 20 patients (11 males, 55.00%), aged 71.38 ± 4.37 (48–80) years. The CG included 19 eyes of 19 patients (10 males, 57.89%), aged 70.31 ± 7.16 (51–79). In the case of TG, 26 eyes had been operated once (68.42%) and 12 eyes had been operated twice (31.57%). The mean interval following the last procedure in the TG was 17.56 ± 2.38 (5–36) months. 

Statistical analysis of findings was performed using SPSS 8.0 (SPSS, Chicago, IL, USA). Statistical significance was set at 0.05. Differences in GAT, DCT, dIOP, CCT, AL, ACD, and age between groups were examined using one-way Analysis of Variance (ANOVA) whereas differences in gender distribution were examined with Pearson's chi square test. Post-hoc analysis of differences between groups was performed with Dunnett's T3 test. The correlations between GAT, DCT, or dIOP and CCT, AL, ACD or patients'age were examined in all groups using Pearson's bivariate correlation coefficient. In the TG, correlations between dIOP and the postoperative interval were also examined using Pearson's bivariate correlation coefficient whereas differences in GAT, DCT, and dIOP between patients having undergone 1 or 2 procedures were examined using independent samples *t*-test. 

## 3. Results

Differences in GAT-IOP, DCT-IOP, age, AL, and ACD between groups were statistically not significant (one-way ANOVA). Differences in gender distribution between groups were also statistically not significant (Pearson's Chi square test). CCT was significantly lower in both TG and LG, compared with CG (Dunnett's T3 test *P* =  .02) whereas differences in CCT between TG and LG were statistically not significant. The dIOP was significantly higher in TG, compared with both CG and LG as well as in LG, compared with CG (one-way ANOVA, Dunnett's T3 test). AL, CCT, ACD, GAT-IOP, DCT-IOP, and dIOP values in all groups as well as ANOVA F values and respective levels of statistical significance are presented in Table 1. 

**Figure 1:**
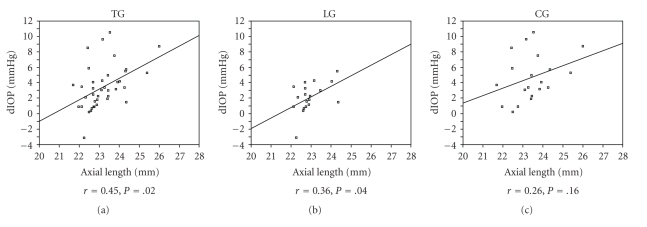
Scattergrams of the correlations between dIOP and AL in the TG (a), LG (b), and CG (c) with respective trend lines, correlation coefficient values and *P*-values.

Among TG patients, dIOP was also significantly higher in patients with a history of 2 procedures, compared with patients with a history of 1 procedure (independent samples *t*-test value 2.46, *P* =  .03). On the contrary, the correlation of dIOP with the postoperative interval from the last procedure was statistically not significant (Pearson's bivariate correlation coefficient). Correlations between dIOP and patients' age, CCT, or ACD were statistically not significant in all groups (Pearson's bivariate correlation coefficient). On the contrary, correlations between AL and dIOP were statistically significant in both TG (Pearson's bivariate correlation coefficient 0.31, *P* =  .01) and LG (Pearson's bivariate correlation coefficient 0.26, *P* =  .03) but not in CG. Scattergrams of the correlations between dIOP and AL in the TG, LG, and CG with respective trend lines, correlation coefficient values and p-values are presented in Figures 1(a), 1(b), and 1(c), respectively.

## 4. Discussion

This study examined dIOP in glaucomatous eyes in which target IOP had been reached following 1 or 2 trabeculectomies (without any medical treatment), in glaucomatous eyes also successfully treated with latanoprost as monotherapy, as well as in a control group of non-glaucomatous eyes. Results imply that dIOP is significantly increased following trabeculectomy which could be related with induced alterations to the biomechanical properties of the ocular walls. 

DCT measurements are produced by a sensortip requiring no applanation of the corneal surface, so they are theoretically not affected by any force-to-pressure translations, as opposed to GAT [9, 10]. Thus DCT measurements may be less dependent on corneal biomechanical factors (especially corneal thickness) than GAT [11], which is also potentially affected by a massaging effect on the aqueous associated with applanation [1]. A previous study has reported increased dIOP values in glaucomatous eyes under monotherapy with latanoprost, compared with glaucomatous eyes under medical treatment with no prostaglandin analogues (PGA) and has concluded that the increase in dIOP may reflect connective tissue remodelling (possibly due to the induction of metalloproteinases by latanoprost) in the ocular walls and thus alterations in their biomechanical properties [1]. Findings from the present study imply that trabeculectomy may also create measurable long-lasting changes in ocular bio-mechanics. Previous studies have stressed the potential effects of the creation of partial thickness flaps on the tectonic properties of ocular walls [5, 6]. In the case of Laser in Situ Keratomileusis (LASIK), the corneal flap is never fully reintegrated into the remaining corneal tissue so it does not participate in its tectonic structure [12, 13]. Furthermore, profound changes in corneal bio-mechanics have been described following LASIK [14]. Undoubtedly, scleral flap creation in trabeculectomy differs in many aspects from corneal flap creation in LASIK, including differences in size and depth, suturing and covering with conjunctiva as well as the posttrabeculectomy sharp decreased in IOP. On the other hand, both cornea and sclera are relatively avascular tissues, implying that long-term adhesion of the partial thickness flaps on their underlying beds may be incomplete. The fact that both corneal flap (in LASIK) and partial thickness scleral flap (in trabeculectomy) can be re-raised in cases of revision of the initial procedure further supports this concept [12–15]. 

In all eyes that necessitated a second trabeculectomy in this study a new flap was created, nasally to the initial flap, instead of revising the site of the initial procedure. This strategy was chosen in an effort to reduce any toxic effects on the sclera at the initial trabeculectomy site, since Mitomycin-C was applied in all secondary procedures, according to previously proposed protocols [16]. The fact that eyes with a history of 2 trabeculectomies displayed higher dIOP, compared with eyes with a history of 1 trabeculectomy, supports the possibility that the added effect of 2 partial thickness scleral flaps may augment any induced alterations in ocular biomechanics. On the other hand, this may also reflect wound healing modification by Mitomycin-C, which was only used in secondary procedures in the present study. Interestingly, dIOP in this study was not correlated with the postoperative interval following the last trabeculectomy. This finding implies that any changes in the elastic behaviour of the ocular walls following trabeculectomy are not continuous but instead are completed at some point (possibly early) along the postoperative course. 

In this study, all eyes of the TG group had in the past been treated with PGA before the first trabeculectomy. PGA use had also been repeated for some time in most eyes with failed trabeculectomies before the decision to proceed to a secondary procedure. Taking into account that latanoprost has been found to significantly increase dIOP [2], the increased dIOP levels in eyes of the TG may, at least in part, also reflect previous PGA use. However, the fact that dIOP was significantly higher in TG compared with LG implies that trabeculectomy may exert added effects on dIOP levels. The duration of latanoprost use has not been significantly associated with dIOP by a previous study [2]. Therefore, any potential differences in the duration of latanoprost use between TG and LG possibly have not affected the recorded difference in dIOP between the 2 groups. 

The fact that AL was significantly correlated with dIOP in both TG and LG may also reflect previous use of PGA in both groups, taking into account that AL (and not CCT) has been previously correlated with latanoprost use [2]. As previously mentioned, this finding may possibly be attributed to remodelling of the scleral collagen or changes in choroidal circulation associated with latanoprost, such as an increase in ciliary body thickness or changes in choroidal vascular permeability [17, 18]. The correlation of AL with dIOP reflects the contribution of both anterior and posterior ocular walls to the total ocular biomechanical behaviour (as opposed to CCT which reflects only its corneal component). Therefore, the fact that the correlation of AL with dIOP was more pronounced for TG, compared with LG, implies that trabeculectomy may affect total ocular rigidity. 

The nonrandomized design and the relatively small number of participants may be considered as weak points for this study. Furthermore, the fact that the postoperative interval on which patients were examined was not stable but instead varied (5–36 months) should be taken into consideration. The prospective consecutive recruitment and fact that all measurements were performed by the same experienced examiner who was masked against patients' classification possibly enhance the validity of results. The dIOP score in the present study was comparable to that reported in previous studies [10, 11] whereas TG, LG, and CG did not differ in patients' age (which has been found to affect dIOP by previous studies [1]). The fact that CCT was significantly lower in both TG and LG (glaucomatous eyes), compared with CG (non-glaucomatous eyes) may be attributed to the inverse correlation between CCT and the predisposition for glaucoma [19]. However, dIOP may be more pronounced only in very thick or very thin corneas [20] (which was not the case for patients in this study) whereas the reported effect of CCT on GAT is statistically weak (*R^2^* ranging from 0.06 to 0.17) [21]. Therefore, we believe that the difference in CCT between groups in this study is unlikely to have affected results. 

There are controversial reports on the effects of glaucoma on ocular rigidity [22–24] as well as the differences in dIOP between treated and untreated glaucomatous eyes [1]. In the present study, the facts that target IOP had been reached in both TG and LG and that NFI did not differ significantly between them imply that glaucoma was equally advanced in both groups. Therefore, the observed differences in dIOP between LG and TG are possibly related with trabeculectomy rather than with the glaucomatous process *per se*. 

Recent evidence suggests that the biomechanics of the corneoscleral shell affect cellular deformation in the optic nerve head, as relatively thick, solid sclera is much stiffer than both neural tissue and the porous lamina cribrosa [25]. Scleral deformation depends on IOP and—more interestingly—on the mechanical properties of the sclera [25]. Findings from this study imply that apart from lowering the IOP trabeculectomy might have additional effects related with changes in scleral biomechanics. Larger randomized studies would be required to further explore this possibility. Results could help in better understanding the pathophysiology underlying ocular rigidity as well as in the design of more powerful procedures for the management of glaucoma. 

## Figures and Tables

**Table 1:** Mean NFI, AL, CCT, ACD, GAT-IOP, DCT-IOP, and dIOP scores in TG, LG, and CG as well as ANOVA F values and respective levels of statistical significance.

Parameter	TG	LG	CG	F	*P*
NFI	42.14	39.28	16.89	2.08	.37
AL (mm)	23.18	22.95	23.01	2.34	.11
CCT (*μ*m)	513.26	510.31	527.84	4.28	.04
ACD (mm)	2.74	2.92	3.03	2.98	.09
GAT-IOP (mmHg)	14.54	15.75	15.11	0.38	.68
DCT-IOP (mmHg)	19.73	19.76	17.09	1.44	.24
dIOP (mmHg)	5.19	4.01	1.98	7.07	.01
